# Coding-Complete Genome Sequence of an African Swine Fever Virus from an Outbreak in 2021 among Domestic Pigs in Pangasinan, Philippines

**DOI:** 10.1128/mra.00719-22

**Published:** 2022-11-09

**Authors:** Andrew D. Montecillo, Zyne Baybay, Ralph Carolyn Cabug, Wreahlen Cariaso, John Paulo Jose, Sheila Mantaring, Annabelle Briones, Amanda Warr, Christine Burkard, Lucille Villegas, Homer Pantua

**Affiliations:** a Microbiology Division, Institute of Biological Sciences, University of the Philippines Los Baños, Los Baños, Philippines; b BioAssets Corporation, City of Santo Tomas, Batangas, Philippines; c Industrial Technology Development Institute, Department of Science and Technology (DOST-ITDI), Bicutan, Taguig City, Philippines; d The Roslin Institute and Royal (Dick) School of Veterinary Studies, University of Edinburgh, United Kingdom; KU Leuven

## Abstract

We report a coding-complete genome sequence of an African swine fever virus from an outbreak in 2021 among domestic pigs in Pangasinan, Philippines using Oxford Nanopore Technologies minION. The linear genome assembly is a single contig with 192,377 bp.

## ANNOUNCEMENT

The first African Swine Fever (ASF) outbreak in the Philippines was recorded in July 2019, and it continues to spread, leading to devastating impacts in the livestock industry. ASF, caused by the sole member of double-stranded DNA virus of the genus *Asfivirus*, family *Asfarviridae*, with a genome size of 170 to 194 kb, is a highly contagious disease with up to 100% fatality among infected domestic and wild pigs (1). Full-genome sequencing of the ASF virus is essential in understanding the biology and epidemiology of the pathogen. Here, we report an ASF virus coding-complete genome locally sequenced using Nanopore sequencing technology.

Extraction, library preparation, and sequencing kits were used following the respective manufacturer’s protocol unless otherwise specified. Whole-blood samples were collected from pigs by a licensed veterinary consultant following the guidelines of the Bureau of Animal Industry, Department of Agriculture (Philippines) during an ASF outbreak in Pangasinan, Philippines in 2021. The total DNA was extracted in duplicate from a frozen ASF diagnostic PCR-positive EDTA-stabilized sample (from a grower pig) using MagMax DNA Multi-Sample Kit (Thermo Fisher Scientific). Then, NEBNext microbiome DNA enrichment kit (New England BioLabs) was used to deplete host DNA.

At least 5 ng of DNA was used as input to the rapid PCR barcoding kit (SQK-RPB004; ONT). The library was loaded on MinION SpotON R9.4.1 FLO-MIN106 flow cell (ONT) and sequenced using the 24-h script in MinKNOW Software (v.22.03.5; ONT) on MinION MK1b device. Reads were basecalled using super accurate model (dna_r9.4.1_450bps_sup.cfg) with default parameters in Guppy v6.0.6 (ONT).

Basecalled reads were combined in one fastq file and were quality-controlled using Porechop v0.2.4 (https://github.com/rrwick/Porechop) with default parameters (-discard_middle). Reads were then mapped using Minimap2 v2.17-r941 (2) (-ax map-ont) against Sscrofa11.1 reference (GCF_000003025.6) (3) to remove host-derived reads. The unmapped reads, extracted with SAMtools v1.9 (4), were used for *de novo* assembly using Flye v2.4.2 (5) (parameters: nano-hq, genome size = 200 k, metagenome mode). The assembly was polished in Medaka v1.4.4 (https://github.com/nanoporetech/medaka) and was aligned against ASFV whole genomes from RefSeq database using MAFFT v.7.490 (6). Maximum likelihood trees were constructed in IQTREE v.2.2.0 (7).

**FIG 1:**
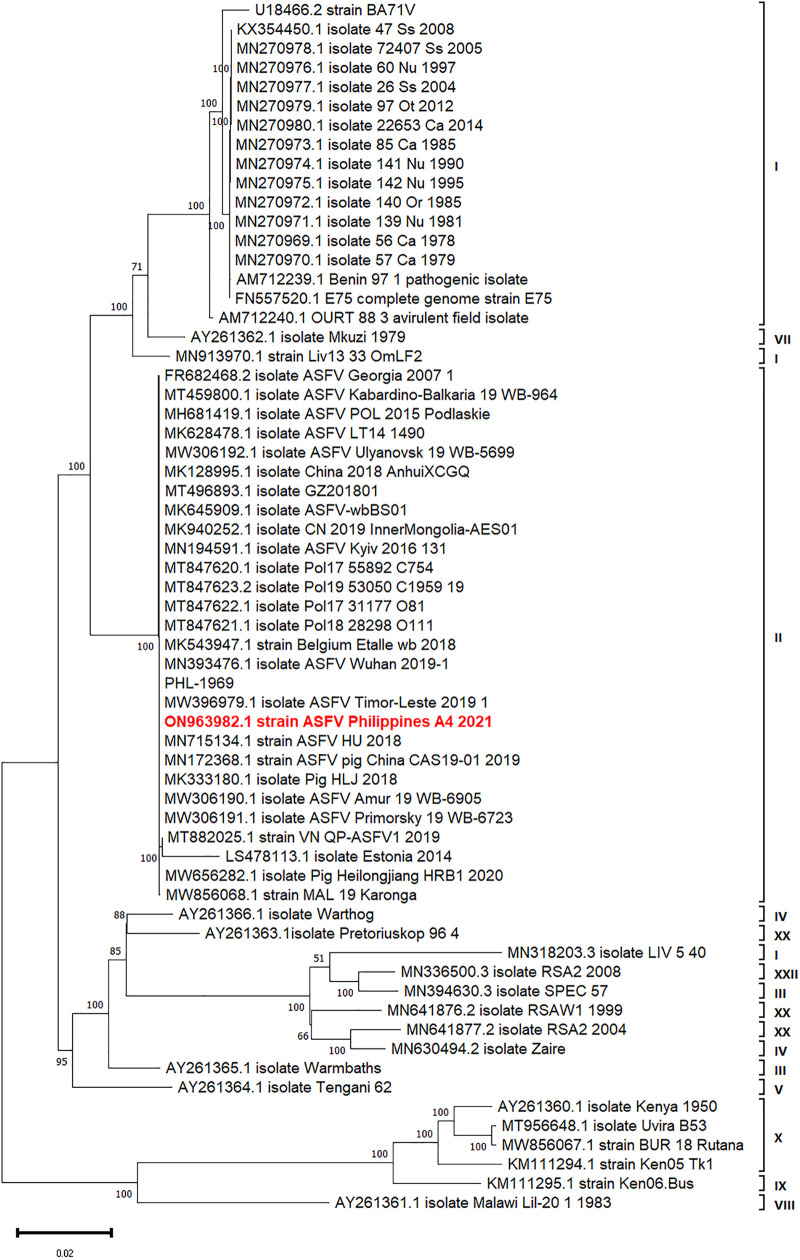
Maximum-likelihood consensus tree of select ASFV reference genomes and Philippines A4 2021 strain (ON963982.1) inferred using the ultrafast bootstrap (9) implemented in the IQ-TREE software (substitution model: GTR+F+R3). The tree is midpoint rooted. The scale bar is given in numbers of substitutions per site, and bootstrap resampling (1,000 replications) support values are shown at the nodes.

A total of 105,660 reads (N50 = 4.23 kb) were generated of which 93,582 were retained after adapter trimming. About 1,422 of these did not map to Sscrofa11.1 reference genome. Mean assembly coverage was 21x (200 kb target genome size) and consisted of three linear contigs (203,823 bp total length). Only the longest fragment (192,377 bp, 38.3% GC content) was confirmed to be ASFV DNA using NCBI blastn search against RefSeq ASFV genomes (taxid:10497) on NCBI Virus database (accessed April 2022). The ASFV contig was aligned against PHL-1969 genome (8) and showed 99.88% identity in the longest contiguous alignment (192,120 bp) with 15 mismatches and 221 gaps but was missing the first 2,300 bp of the PHL-1969 genome 5′-end. Our strain belongs to the clade with PHL-1969, Timor-Leste 2019 strain (MW396979.1), Wuhan 2019 (MN393476.1), and other genotype II strains from China, Ukraine, Belgium, and Poland (Fig. 1).

### Data availability.

The genome sequence has been deposited in NCBI GenBank under the accession no. ON963982, and the raw sequence data can be found in the GenBank SRA under BioProject accession no. PRJNA857442. The version described in this paper is the first version, ON963982.1.
